# The Maxims of John Arderne, Master Surgeon

**Published:** 1910-11-05

**Authors:** 


### Medical Antiquities.

### THE MAXIMS OF JOHN ARDERNE, MASTER-SURGEON.

There have been many rules of life written in different ages for or by the physician, for his own guidance, or that of his disciples or readers; they vary within the widest limits of tone and atmosphere, from the epic grandeur of the "Religio Medici" to the cynical brutality of "Count von Fotham": each and all of them, however, clothed in that perennial interest which is inherent in the subject itself, no matter how treated. It is with pleasure, therefore, that we draw attention to the maxims of John Arderne, Master-Surgeon to John of Gaunt, "Duc de Lancastre, Roy de Castell, et de Leon, Monseigneur d'Espagne,"* representing, as they do, the code of ethics held by the highest representatives of the art in the fourteenth century, and casting, likewise, some very curious sidelights on the status of the "Leech," then a vassal in the train of one or other of the great feudal princes.

### Arderne's Life and Work.

John Arderne was born in 1307, and saw much service against the Moors in Spain, and with the armies of the Black Prince during the earlier and more brilliant period of the Hundred Years' War. He practised at Newark from 1340 to 1370, and wrote his work on "Fistula in Ano" in 1376. In this work, following the example of William of Salicet and Henri de Mandeville, he incorporated the maxims to which we shall now refer, and which may be entitled the whole duty of a surgeon. First of all, with the characteristic piety which is so pronouncedly mediæval in its open expression, he commences his injunctions thus: "it behoveth him that will profit in this craft that he set God before him evermore in all his works, and evermore call meekly with heart and mouth (for) his help," adding that it is the duty of the leech to be charitable in gratuitous treatment of the poor, "that they by their prayers may get him grace of the Holy Ghost."

### The Ideal Doctor.

Next, descending to particulars, he occupies himself with the manners of the leech. He must be modest, "not temerarie or boastful in his sayings or in his deeds" like the "quacks," who get their name from their much talking which signifieth nothing. On the contrary, he is advised to be quiet in his manner, abstaining from much speech, especially when in the company of great men. The manner of the leech should be sedate, he should not be given to "much laughing or much playing." His friends should be carefully chosen, so that "as much as he may without harm should he flee from the fellowship of knaves." He should not be content to give his attention to his calling solely when necessity demands it, but all the time he should be studious, "occupied in the things that beholdeth to his craft, either read he or study he, or write or pray he; for the exercise of books worshippeth a leech. For why (*i.e.* because) he shall both be beholden (wise), and he shall be more wise."

### Moral Precepts.

In days when gross eating and gross drinking were the commonplaces of domestic life he strikes a curiously modern note when he adds that the leech should be sober, "for above all these it profiteth to him that he be founden sober; for drunkenness destroyeth all virtue, and bringeth it to nought." He adds that gluttony is contemptible, and that the leech "should be content in strange places of meats and drinks there y-founden, using measure in all things." His next note on the evils of professional jealousy might have been written to-day: "Scorn he no man, for of that it is said he that scorneth other men shall not go away unscorned (*Deridens alios, non inderisus abibit*)." And his advice, when the patient or his friends, probably unnecessarily, suggest bringing in some brother practitioner, of whom he knows little, in consultation, could not possibly be improved on: "If there be made speech to him of any (other) leech neither set he him at nought nor praise him too much or commend him. But this may he courteously answer: 'I have not true knowledge of him, but I learned nought (against him), nor I have not heard of him (anything) but (what is) good and honest.' And of this shall honour and thankings of each party (the patient and the other leech) increase and multiply to him. After this honour is in the honourant and not in the honoured."

### More Moral Precepts.

Then follows some advice on chastity and continence which casts a lurid sidelight on the morals of mediæval times; and this is succeeded by a warning not to fall out with the servants around the great. Nevertheless, the leech had to maintain the dignity of his high calling, and so, though advised not to be overbearing, he is likewise warned against the opposite pitfall of over-familiarity, "for too much homeliness breedeth dispising." The next healing introduces a sinister note, now, happily, extinct. Men's minds in those days were very suspicious, owing to ignorance. Many a natural death was, therefore, ascribed to poison, and too often the leech in attendance was supposed to have been suborned by an enemy to administer the alleged poison. Failure even to cure some particular ailment sometimes brought dire results, as witness the case of John of Bohemia, from whom the feathers in the arms of the Prince of Wales are derived, who had his leech sewn in a sack and then thrown into the Seine, after a course of unsuccessful treatment. Arderne can, therefore, only be accused of caution when he advises in what appears difficult or incurable cases to avoid treatment, inventing some diplomatic excuse "that he may not incline to their askings," and yet, at the same time, not cause "indignation of (in) some great man, or his friend," by such refusal. If nothing else avails, he therefore advises that the leech should "feign he him (*i.e.* himself) hurt, or that he was sick (ill), or some other convenable cause by which he may likely be excused."

If, however, he does decide to treat the case, he must make "covenant for his travaile, and take it beforehand," for then, as now, patients had a habit of forgetting to pay the surgeon his just dues when they were healed. If he decides to operate, he is advised to state his fee beforehand, and not to ask too little, "ever be he wary of scarce askings, for over-scarce askings setteth at nought both the market and the thing," or in other words, patients then, as now, appreciate the surgeon, and his skill in operating on them, in proportion to the fee they have had to give for his services.

The Need for Caution in Prognosis.

Again, his mediæval caution peeps out when he advises the leech not to promise a cure too soon, "for it is better that the term be lengthened, than the cure, for prolongation of the (promised time of) cure giveth cause of despairing to the patient," and he loses confidence in his surgeon; but if he recovers before the allotted time, then is there so much the more joy, and the patient both takes credit for his good constitution, and praises the surgeon for his superior skill. Next follows advice as to the surgeon's dress. "His clothes and other apparelling should be honest, not liking himself in apparelling or bearing to minstrels, but in clothing and bearing shew he the manner of Clerks (Clergymen)." His person should be scrupulously clean, and his manner quiet, "and when he shall speak be the words short, and, as much as he may, fair and reasonable, and without swearing." He should be of a cheerful countenance in the sick room, and have a fund of tales "that may make the patients to laugh." Finally, he is bound to secrecy as to anything he may have learnt in his professional capacity of the patients' secrets. "Discover never the leech the counsels of his patients, whether of men or women." These thing, if he will observe, though they are but a few of the many, yet if kept "they will give a gracious going to the user" so that he may attain "to the height of worship and of winning."

* "Treatises of Fistula in Ano, Hæmorrhoids and Clysters, by John Arderne." Edited by D'Arcy Power, F.R.C.S. Early English Text Society. 1910.

